# Physiological Diversity of Spitsbergen Soil Microbial Communities Suggests Their Potential as Plant Growth-Promoting Bacteria

**DOI:** 10.3390/ijms20051207

**Published:** 2019-03-09

**Authors:** Agnieszka Hanaka, Ewa Ozimek, Małgorzata Majewska, Anna Rysiak, Jolanta Jaroszuk-Ściseł

**Affiliations:** 1 Department of Plant Physiology, Faculty of Biology and Biotechnology, Maria Curie-Skłodowska University, Akademicka St. 19, 20-033 Lublin, Poland; 2 Department of Environmental Microbiology, Faculty of Biology and Biotechnology, Maria Curie-Skłodowska University, Akademicka St. 19, 20-033 Lublin, Poland; ozimek@poczta.umcs.lublin.pl (E.O.); majewska@poczta.umcs.lublin.pl (M.M.); jolanta.jaroszuk-scisel@poczta.umcs.lublin.pl (J.J.-Ś.); 3 Department of Ecology, Faculty of Biology and Biotechnology, Maria Curie-Skłodowska University, Akademicka St. 19, 20-033 Lublin, Poland; anrysiak@poczta.umcs.lublin.pl

**Keywords:** ACC deaminase, IAA, PGPB, phosphate solubilization, *Phaseolus coccineus*, rhizosphere, root length, seed germination, siderophore

## Abstract

The objective of the study was to assess the physiological diversity and metabolic activity of the soil bacterial communities inhabiting Spitsbergen soils in search of bacterial abilities facilitating plant growth promotion. In the soil, the total number of culturable microorganisms, the number of their individual physiological groups (including Siderophore Synthesizing; SSB and Phosphate Solubilizing Bacteria; PSB), the dehydrogenase (DH) activity, and the ability to utilize sources of C, N, P (EcoPlate) were analysed. In bacterial isolates, siderophores production, ACC (1-aminocyclopropane-1-carboxylate) deaminase (ACCD) activity, IAA (indole-3-acetic acid) synthesis were examined. The isolates were applied to the seeds of *Phaseolus coccineus* regarding their germination and root length. The results showed differences between copio- and oligotrophic bacteria. A usually high number of SSB was accompanied by the raised number of PSB. A bigger number of SSB was connected with low values of Fe in the soil. High DH activity was assisted by greater number of copio- and oligotrophic bacteria, raised average well color development value, and N and C contents in the soil. Germination index was more alike relative seed germination than relative root growth. IAA concentration and ACCD activity were conversely related. Synthesis of siderophores was matched with ACCD activity and its high level was combined with elevated germination index. In spite of different localization of soil samples, some isolates proved similar traits of activity. Distinct affiliation of isolates and their various localizations were displayed. Among all isolates tested, some possessed one main trait of activity, but most of them had two or more significant features for potential plant growth stimulation. These isolates could be an important source of useful bacteria.

## 1. Introduction

Plants are associated with a large diversity of microorganisms that are defined as the plant microbiota. The rhizosphere microorganisms, especially viable and culturable, act in plant physiology as growth stimulators, nutrient suppliers, pathogenesis inhibitors, and soil structure improvers [1,2,3]. Plant growth promoting bacteria (PGPB) are able to improve plant performance and systemic resistance, especially under stress conditions [4,5,6], using a wide variety of direct and indirect mechanisms, e.g., biological nitrogen fixation, siderophore production (SSB; Siderophore Synthesizing Bacteria), minerals solubilization, synthesis of enzymes, generation of phytohormones, and facilitating uptake of nutrients from the soil [7,8].

Siderophores, low-molecular-weight organic compounds capable of chelating iron, are able to sequester Fe from the soil and provide it to a plant as a bacterial siderophore-Fe complex or in an exchange reaction using an appropriate ligand [4,9]. Insoluble minerals, such as phosphorus, are solubilized by 1–50% of the population of rhizospheric bacteria [10]. Phosphate solubilizing bacteria (PSB) convert the phosphates into available forms through acidification, chelation, exchange reactions, release of complexing, or mineral dissolving compounds (e.g., organic acids), among other [10,11]. 

Microbial ACC (1-aminocyclopropane-1-carboxylate) deaminase (ACCD), the ethylene (ET) precursor, cleaves ACC (exuded by plant tissues and then taken up by the bacteria), lowering the ET level in the plant [3,8,12,13]. Well-known cross-talk between phytohormones and metabolites of plant resistance induction pathways, auxin, indole-3-acetic acid (IAA), and ET [14], is based on activation of ACC synthase by IAA, which leads to production of ACC and subsequently ET [15,16]. ET has a special significance in resistance to cold stress. PGPB lowers ET concentration to a level that does not inhibit plant growth, and simultaneously it can still effectively serve as a signal molecule in ET/jasmonic acid (JA)-dependent induced systemic resistance [17].

Tryptophan (Trp), present in root exudates, is the main precursor in five different pathways of IAA biosynthesis in bacteria: indole-3-acetamide (IAM), indole-3-pyruvic acid (IPyA), indole-3-acetonitrile (IAN), tryptamine (TAM), and tryptophan side-chain oxidase (TSO) pathways [18]. The existence of Trp-dependent and independent pathways was also reported in bacteria [6,19,20] and plants [21]. Bacterial IAA production is modulated by plant extracts, specific compounds in plant surfaces, and environmental stresses (such as carbon limitation, acidic pH, and osmotic and matrix stress) interfere with all the processes in plants, which are controlled by IAA (e.g., cell division and enlargement, tissue differentiation, light and gravity response) [13]. 

Considering the natural biodiversity of PGPB, the number of formulations used for producing inoculants for plants is relatively low and calculated for around 30 genera and 60 species, such as *Achromobacter* sp., *Alcaligenes* sp., *Burkholderia* sp., *Ochrobactrum* sp., *Pseudomonas* sp., *Pantoea* sp., *Serratia* sp., and *Stenotrophomonas* sp. [7,22].

The status and productivity of the soil system depends upon the structure and functions of soil microbial communities [23,24]. The soil environment of Spitsbergen is dynamic and variable against the severe climate conditions [25]. In the face of changing environmental conditions, microorganisms can rapidly modify their biomass, metabolic activity, and community structure and diversity due to high adaptability and plasticity [26]. 

Meteorological data of Spitsbergen predict continued climate warming [25], which could lead to decomposition of the previously-frozen stores of organic carbon, mainly driven by microbial communities [2]. Permafrost microorganisms are able to grow not only in cold temperatures, but also at 20 °C, which indicates that they are not mainly psychrophiles, specialists with a temperature-restricted niche, but rather psychrotolerant microorganisms [2,25]. The physiological diversity of Spitsbergen soil bacteria is limited to a few studies [27].

The key parameter for evaluation of isolates ability in plant growth promotion, especially in stress condition, is seed germination test and root length [28].

We hypothesize that Spitsbergen soils are biologically active and are the source of isolates, which possess traits that potentially promote plant growth. We question whether the only source of PGPB is soils of the highest biological activity.

The objective of the study was to assess the physiological diversity and metabolic activity of the soil bacterial communities inhabiting Spitsbergen soils in search of bacterial abilities that might facilitate plant growth promotion. PGPB were searched for in soil of high biological activity, which consisted of the total number of culturable microorganisms (copio- and oligotrophic bacteria), the number of their individual physiological groups (SSB; PSB; cellulolytic, CB; amylolytic, AB; ureolytic bacteria, UB), the ability to utilize sources of C, N, P (EcoPlate), and the enzymatic activity (dehydrogenase; DH). In order to estimate activity of bacterial isolate traits directly associated with plant growth promotion were studied, e.g., siderophores production, ACCD activity, and IAA synthesis. Additionally, a pilot research was conducted to investigate the direct effect of isolates on a model dicotyledonous plant, *Phaseolus coccineus* L., using germination test. In order to achieve the goal set, analyses of soil and isolates were compared.

## 2. Results

### 2.1. Microbiological Characteristics of the Soil Samples

#### 2.1.1. Number of Heterotrophic Bacteria and Bacteria with Lytic Activity

**Figure 1:**
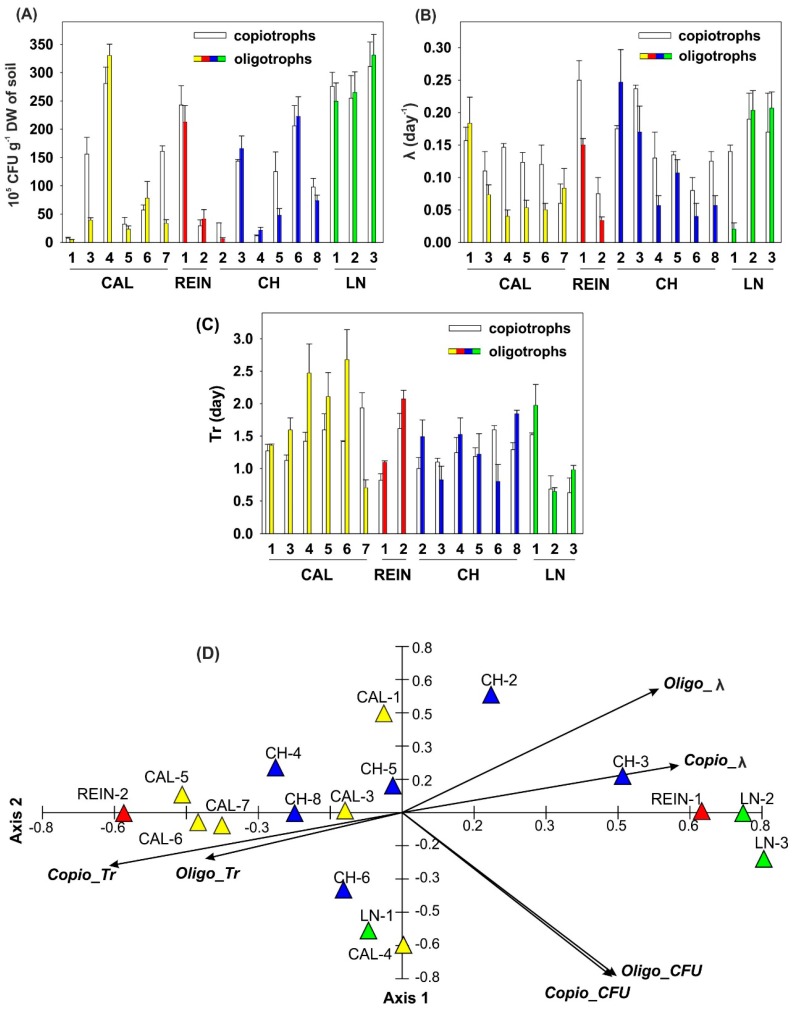
The colony formation by copiotrophic and oligotrophic soil bacteria based on the First Order Reaction (FOR) probabilistic model proposed by Hattori (1985), number of copiotrophic and oligotrophic bacteria; colony-forming unit (CFU) (**A**); λ = probability of visible colony formation per unit time (**B**); Tr = retardation time (**C**). The principal component analysis (PCA) for number of copiotrophic (Copio) and oligotrophic (Oligo) bacteria, and their λ and Tr (**D**).

Above 2 × 10^7^ colony-forming unit (CFU) g^−1^ dry weight (DW) of soil copio- and oligotrophic bacteria were demonstrated in 6 soil samples, CAL-4, REIN-1, CH-6, and all LN, being the highest for the first mentioned sample (Figure 1A). The lowest number of bacteria (below 1 × 10^7^ CFU g^−1^ DW of soil) was determined for 7 out of 17 soil samples in copiotrophs and for 10 samples in oligotrophs. Above 2-fold difference between the number of copio- and oligotrophic bacteria was confirmed in 4 soil samples (CAL-1, CAL-7, CH-2, and CH-5). The most elevated λ values, > 0.23 day^−1^, were detected in REIN-1 and CH-2 for oligo- and copiotrophs, respectively (Figure 1B). Low λ values (≤ 0.05 day^−1^) were approved for 5 oligotrophic bacteria samples with the lowest value of 0.02 day^−1^ in LN-1 sample. Above 2-fold difference between CFU of both tested bacterial groups was determined in the samples: CAL-4, CAL-5, CAL-6, REIN-2, CH-4, CH-8, and LN-1. Tr > 1.5 was demonstrated for 5 and 7 samples of oligo- and copiotrophs, respectively (Figure 1C). For copiotrophic bacteria, the highest values were in CAL-7, REIN-2, and CH-6, and for oligotrophic bacteria it was CAL-4 and CAL-6. Two-times or higher difference between the Tr of copio- and oligotrophic bacteria was confirmed in 2 soil samples, CAL-7 and CH-6.

The first two axes of the principal component analysis (PCA) based on First Order Reaction (FOR) model explained 78.2% of data variability, including the first one with 51.8% (Figure 1D). The number of oligotrophic (Oligo_CFU) and copiotrophic (Copio_CFU) bacteria was positively correlated with axis 1 and λ values for copio- and oligotrophic bacteria (Copio_λ and Oligo_λ, respectively), and were most strongly associated with the discussed axis. Retardation time for copio- and oligotrophic bacteria (Copio_Tr and Oligo_Tr, respectively) was negatively related with axis 1. All variables of this analysis were negatively correlated with the second axis of the ordination diagram, with the exception of Copio_λ and Oligo_λ. The numbers of bacteria were strongly interconnected with each other. The highest number of bacteria of both groups was obtained from soils originating from LN-3, CAL-4, LN-2, LN-1, and CH-6. The smallest number of bacteria was grown from soils derived from CAL-7 and 5, CH-4 and 2, and CAL-1. Values of Copio_λ and Copio_Tr were inversely proportional to each other. The high values of Copio_λ for CH-6 and 8, CAL-7, and LN-1 and 3 corresponded to the lowest Copio_Tr values for these locations. Such a clear correlation of the λ and Tr indices was not observed for oligotrophic bacteria. The high value of Oligo_λ for LN-3 and 2 was associated with the high number of bacteria, and at the same time one of the lowest for CH-2 soil.

**Figure 2:**
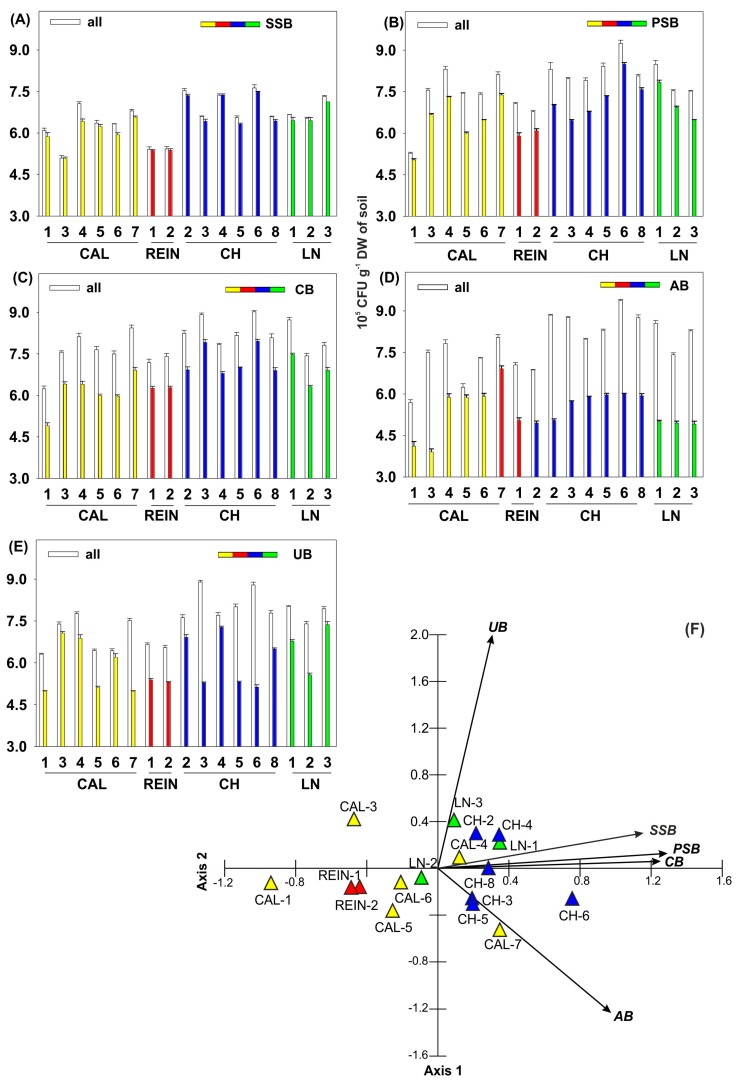
The number of: SSB (Siderophore Synthesizing Bacteria) (**A**); PSB (Phosphate Solubilizing Bacteria) (**B**); CMB (Cellulolytic Bacteria) (**C**); AB (Amylolytic Bacteria) (**D**); UB (Ureolytic Bacteria) (**E**). PCA for SSB, PSB, CMB, AB, and UB (**F**).

The SSB and PSB were detected in all of the analyzed soil samples (Figure 2A,B). Overall, the number of SB was lower than PSB, except for 5 soil samples, CAL-1, CAL-5, CH-2, CH-4, and LN-3. Moreover, CB, AB, and UB were displayed in all soil samples (Figure 2C–E). The number of CB was higher than AB, except similar values were present in CAL-7. The number of UB was the most diversified, being higher, lower, or between values of colony forming units determined for CB or AB.

The first two axes of the PCA diagram presenting specific substrates available in agar plate tests explained about 76.7% of the variability of the analyzed data, including the first axis accounting for about 52.7% (Figure 2F). All variables were positively correlated with the discussed axis. The greatest correlation was demonstrated among the number of PSB, CB, and SSB. For the second axis, statistical significance was shown in the number of UB, SSB, and AB. UB represented the strongest positive correlation with the second axis, SSB was slightly weaker, and AB showed strong negative correlation. The widest spectrum of bacterial activity was evidenced for the CH-6 soil, for 4 (CB, AB, PSB, SSB) of the 5 analyzed bacteria types. A slightly narrower spectrum of activity was noted in CH-5 (CB, AB, PSB), CH-2 (CB, UB, SSB), and CAL-7 (AB, PSB, SSB) soils, showing a large number of bacteria in 3 out of 5 specific media used. The weakest spectrum of bacterial activity was recorded for CAL-1 and 5, and REIN-2 and 1. Moreover, the lowest number of bacteria was found in comparison with the soil from other locations. The numbers of SSBs, PSBs, and CBs were correlated with one another, as indicated by the location of the vectors on the ordination diagram. The numbers of UB and AB were not related with each other and showed little association with the above-mentioned groups of bacteria. The highest numbers of CB, AB, PSB, and SSB were obtained from the soil collected at the CH-6 location. The numbers of PSB and AB were comparable. In the soils from CAL-7, CH-6, 8, and 5, and LN-1, the highest numbers of all specific bacteria were obtained in comparison with other soil samples. The high number of SSB was also found in the soil from CH-4 and 2, LN-3, and CAL-7. The number of UB was the highest for the soil collected from LN-3, CH-4 and 2, and CAL- 3 and 4. The most elevated number of PSB was also stated for CH-8 and 5, LN-1, and CAL-7. In the soil from CAL-6, high activity of only AB was observed, whereas from CAL-3 and 4, it was the same for UB. CAL-6, 3, and 4 soils showed a large selectivity according to the prevailing bacterial activity being only AB or UB dominant.

#### 2.1.2. Determination of Microbial Activity and Catabolic Diversity

The rates of substrate utilization evaluated by Biolog EcoPlates showed that microbial communities were metabolically diversified in the carbon source oxidation (Figure S1). Some of the substrates were widely used as nutrients by soil bacteria, and the rates of their utilization varied according to the total activity of the relevant bacterial community, e.g., d-mannitol, Tween 40, Tween 80, d-cellobiose, pyruvic acid methyl ester, l-asparagine, *N*-acetyl-d-glucosamine, d-xylose, and galacturonic acid. Reduced metabolic activity was established for the following substrates: α-cyclodextrin, glucose-1-phosphate, 2-hydroxy-benzoic acid, d,l-α-glycerol-phosphate, and phenylethylamine.

**Figure 3:**
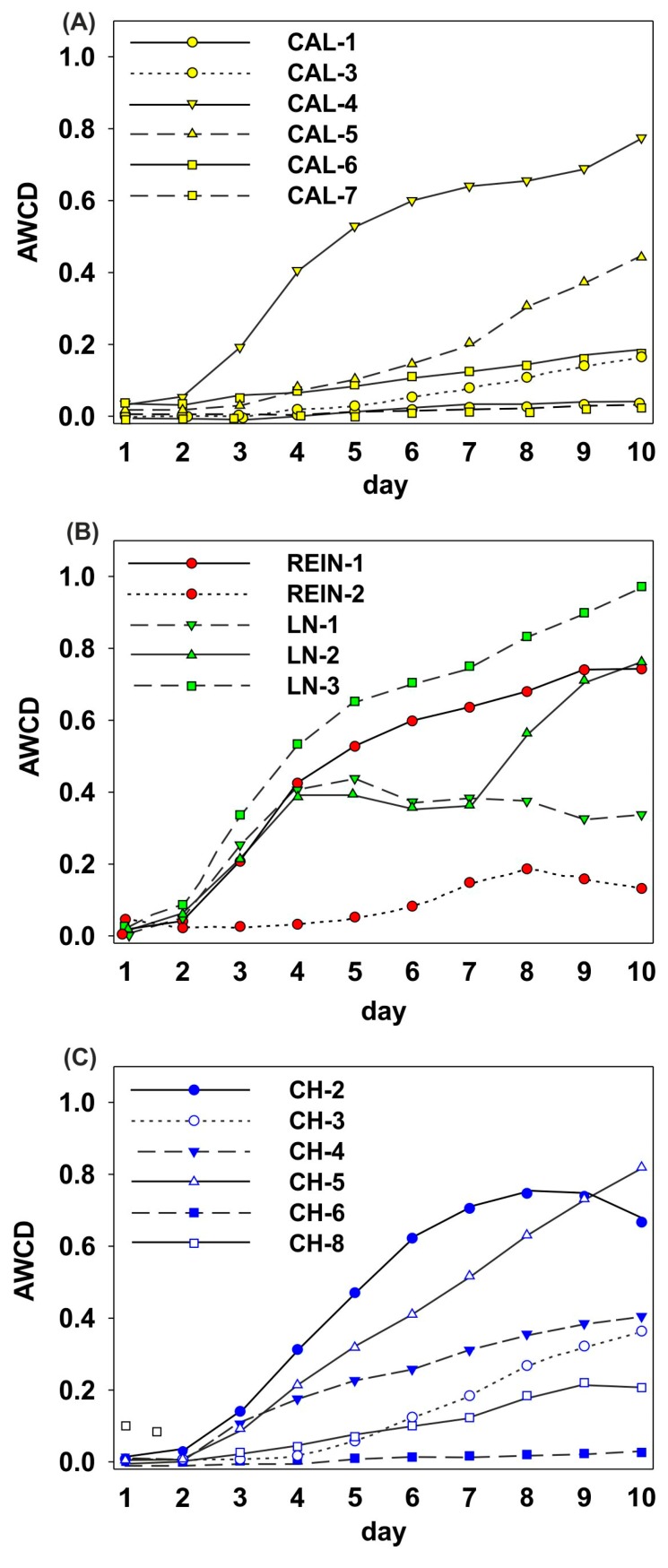
The kinetic curves of AWCD (average well color development) for different soil microbial communities (**A**−**C**).

The kinetic curves of the average well color development (AWCD) (Figure 3) illustrated different patterns of substrate utilization. Three soil samples showed that C utilization by microbial communities stayed almost steady within the measured period (CAL-1, CAL-7, CH-6). Steady progress in AWCD values was proven to be small in two soils, CAL-3 and CAL-6, or rapid in CH-5. Elevation in C utilization with stabilization at the end of incubation time was detected to be small in REIN-2 and CH-8, bigger in CH-4, or the most prominent in REIN-1 and CH-2. There was a short lag phase of color development and sigmoid curves in CAL-4, LN-3, and a longer lag phase in CAL-5 and CH-3. There were two curves of no-well defined course in LN-1 and LN-2.

**Figure 4:**
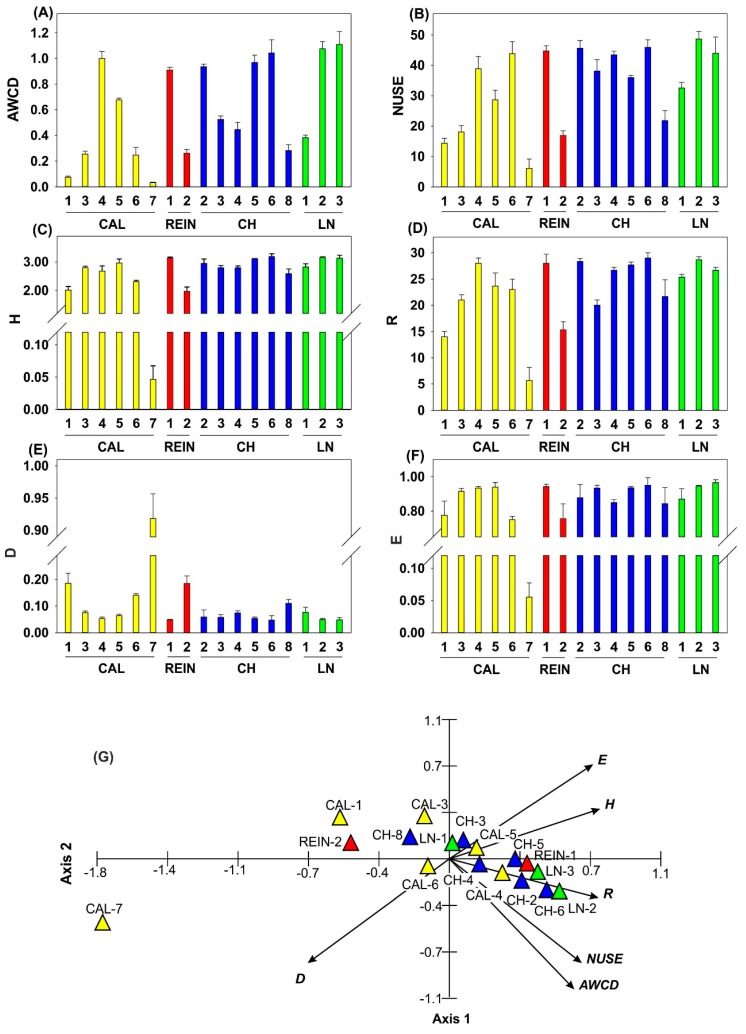
Parameters describing the activity and catabolic diversity of the soil microbial communities: AWCD (**A**); NUSE (nitrogen use index) (**B**); H (Shannon’s diversity index) (**C**); R (substrate richness) (**D**); D (domination index) (**E**); E (substrate evenness) (**F**). PCA for AWCD, NUSE, H, R, D, and E (**G**).

In order to compare the metabolic potential and catabolic diversity in soil samples, 6 indicators were used: AWCD, nitrogen use index (NUSE), Shannon’s diversity index (H), substrate richness (R), domination index (D), and substrate evenness (E) (Figure 4). AWCD values were the most elevated in CAL-4, CH-6, LN-2, and LN-3 (Figure 4A), while NUSE was most elevated in LN-2. All the indicators showed that CAL-1, CAL-7, and REIN-2 had the most protrusive values, the lowest values for AWCD, H, NUSE, R, and E, and the highest for D. Values of D were conversely proportional to E. For 5 indicators, AWCD, H, NUSE, R, and E, the ultimate values were achieved in CAL-4, REIN-1, CH-6, LN-2, and LN-3.

The first two axes of the PCA ordination analysis for Ecoplate parameters showed statistical significance and explained 94.7% of the variability of data, including the first axis of 82.4% (Figure 4G). The analyzed variables were positively correlated with the first axis, with the exception of D, with the strongest correlation depicted by H, R, and E. Most variables were negatively related with axis 2, but AWCD, D, and NUSE were most strongly related. The strongest positive correlation with axis 2 was shown by E, followed by H. Parameters of AWCD, NUSE, and H were correlated with each other, and were simultaneously related to R and E indices. Parameter D was negatively interconnected with both axes and was inversely proportional to the variables discussed earlier. The first axis of the diagram clearly showed the increasing gradient of AWCD, H, and D. On the left side of the diagram there were soils (CAL-7, 1, and 6, and REIN-2), which showed the lowest AWCD and NUSE values, and had the highest D values. The right side of the diagram focused on soils with the highest microbiological potential and simultaneously with the smallest values of D parameter, e.g., LN-3 and 2, CH-4 and 5, and CAL-4.

#### 2.1.3. Enzymatic Activity

**Figure 5:**
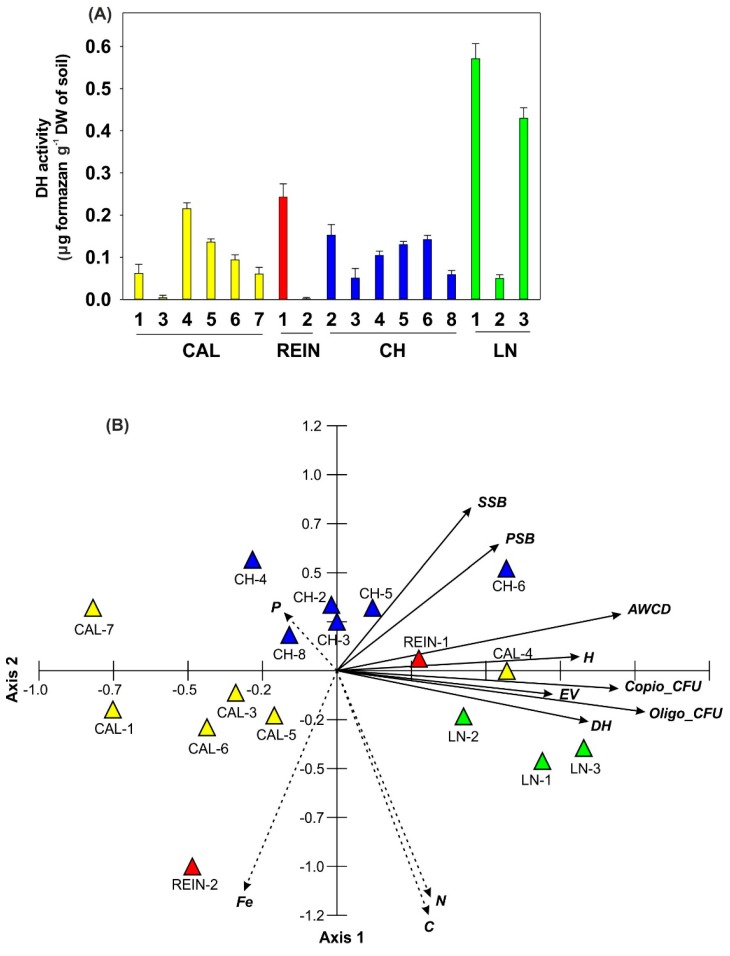
Soil dehydrogenase (DH) activity (**A**). PCA of the soil parameters: number of Copio, Oligo, SSB, and PSB, AWCD index, H index, and DH activity, and concentration of C, N, P, and Fe (**B**).

DH activity in the soil samples ranged from 0.002 to 0.57 µg formazan g^−1^ DW of soil (Figure 5A). Eight soil samples represented activity not exceeding 0.1, and four samples showed higher values than 0.2 µg formazan g^−1^ DW (CAL-4, REIN-1, LN-1, and LN-3).

#### 2.1.4. Relationship among Microbial Parameters Measured for Soil Samples

The PCA analysis showed the relationship among Copio_CFU, Oligo_CFU, SSB and PSB, AWCD, H and E parameters, DH activity, and the content of selected elements (P, N, C, and Fe) in the soils (Figure 5B). The first two axes explained 57.1% of data variability, including axis 1 explaining 33.4%. The analyzed variables were statistically significant (*p* < 0.05), except for the P content for the first axis of the diagram, and Copio_CFU and H for the second axis. Copio_CFU, Oligo_CFU, and AWCD were the most strongly positively correlated with the first axis and with each other. The remaining variables were less positively related with the first axis, except the Fe content, which was negatively related to the discussed axis. Most variables were negatively correlated with the second axis of the diagram, except for the number of specific bacteria, PSB, SSB, AWCD index, and P content, which were positively correlated with it. The first axis of the PCA diagram showed the increasing gradient of the number of oligo- and copiotrophic bacteria, SSB, PSB, AWCD index, and concentration of C, N, P, and Fe. The left side of the diagram focused on the soils (CAL-1, CH-2 and 4, and Cal-5, and 7) with a small number of copio- and oligotrophic bacteria. It was connected with a high content of P, especially for CH-2 and 4, and Fe for CAL-1 and 5. The central part of the diagram collected the locations with the average number of bacteria. On the right side of the diagram, the soils (LN-3, CAL-4, LN-2 and 1, and CH-6) were characterized by the highest number of copio- and oligotrophic bacteria. This was accompanied by the high DH activity, high AWCD value, and high N and C contents in the soil. The content of P in the soil with a high number of microorganisms was floating, was relatively high for LN-3, and the lowest for CAL-4 from all studied soils. The numbers of SSP and PSB were also correlated, and their high values were connected with relatively high Oligo_CFU and high AWCD values for the soils from CH-6 and LN-1.

On the basis of the data achieved for soil samples, 6 of the samples, namely CAL-1, CAL-7, REIN-2, CH-2, CH-6, and LN-1, whose activity seemed to be less pronounced, were rejected from further analysis. Out of remaining 11 soil samples, 29 bacteria were isolated.

### 2.2. Characteristics of Bacterial Isolates

#### 2.2.1. Enzymatic Activity

**Figure 6:**
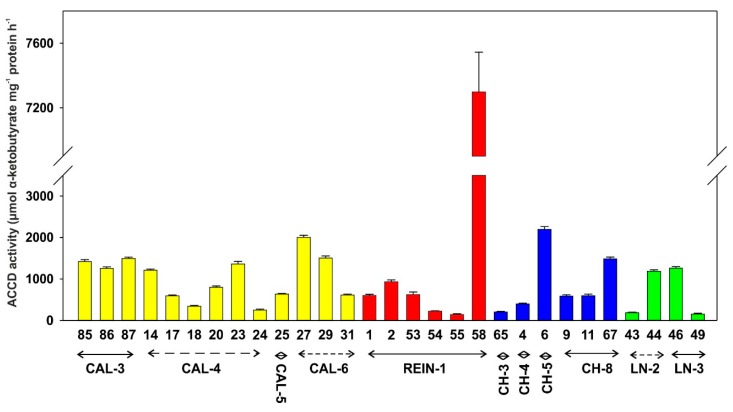
ACCD (1-aminocyclopropane-1-carboxylate deaminase) activity. For other abbreviations see Table 1.

ACCD activity (Figure 6) was the lowest in isolate number 55 and the highest in isolate number 58. Exactly 17 of the isolates displayed activity lower than 1000 nmol α-ketobutyrate mg^−1^ protein h^−1^, and 9 were higher. High activities were demonstrated by isolate numbers 27 and 6.

#### 2.2.2. Synthesis of Growth Regulator

**Figure 7:**
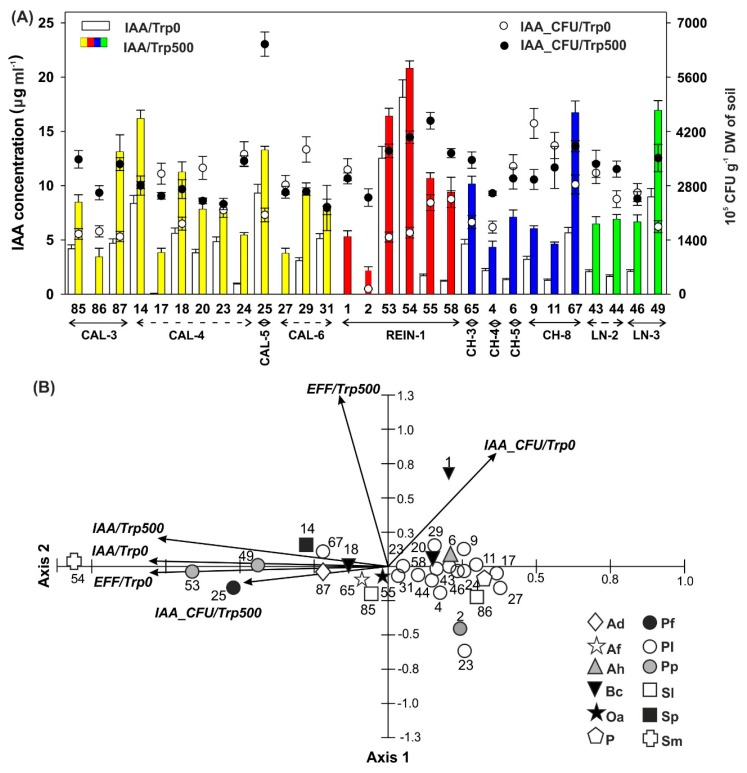
IAA (indole-3-acetic acid) concentration; Trp0 = without tryptophan (Trp); Trp500 = with 500 µg mL^−1^ Trp; number of bacteria producing IAA without and with Trp (IAA_CFU/Trp0 and IAA_CFU/Trp500, respectively) (**A**). PCA for IAA concentration without and with Trp (IAA/Trp0 and IAA/Trp500, respectively), number of bacteria producing IAA without and with Trp (IAA_CFU/Trp0 and IAA_CFU/Trp500, respectively), and efficiency of IAA production without and with Trp (EFF/Trp0 and EFF/Trp500, respectively) (**B**). For other abbreviations, see Table 1.

IAA production (Figure 7) without external Trp supplementation was shown in almost all the isolates, except isolates numbers 86, 27, 1, and 2. The most elevated IAA concentration was in isolate number 54. Without (Trp0) and with addition of 500 µg Trp (Trp500), the highest concentration of IAA was observed for the same isolate, number 55. Directly 23 isolates achieved values higher than 5 µg mL^−1^. In most of the cases, CFU was elevated after Trp implementation.

IAA activity was examined on the basis of 6 variables using PCA; the analysis explained 72.3% of the variability of data for the two axes of the diagram, of which 54.1% was for the first axis. All variables were statistically significant for the first axis of the diagram and mostly negatively correlated with it, with exception of IAA_CFU/Trp0 showing a positive correlation with the discussed axis. In the case of the second axis, three variables (IAA_CFU/Trp0, EFF/Trp500, and IAA/Trp500) were statistically significant and positively correlated with it. The first axis of the diagram showed the increasing gradient of IAA_CFU/Trp500. The right side of the diagram concentrated isolates with low values of this variable, e.g., 2, 86, 27, 17, and 1. The left side of the diagram shows the maximum values of this variable for the isolates 54, 53, 25, 49, and 14. The high values of IAA_CFU/Trp500 were associated with low values of IAA_CFU/Trp0. Mutual positive correlation was demonstrated by IAA/Trp500 and IAA/Trp0. High values of one indicator were associated with the increase of the other, which also positively affected the values of IAA_CFU/Trp500 in isolates 54, 53, 25, and 49. The previously mentioned isolates 49, 87, and 14 were also characterized by high EFF/Trp0 and EFF/Trp500 values.

#### 2.2.3. Minimal Inhibitory Concentration (MIC) for Cu

For 25 of the isolates, MIC for Cu was detected at 10 mM, except for 4 isolates with numbers 2, 4, 24, and 54, which was at 5 mM.

#### 2.2.4. Biochemical Identification of Bacterial Species and Their Ability in Siderophore Synthesis

On the basis of the biochemical analytical profile index (API) tests conducted for 29 isolates obtained from 11 soil samples, the following bacteria were identified: *Achromobacter*, *Alcaligenes*, *Burkholderia*, *Ochrobactrum*, *Pseudomonas*, *Pantoea*, *Serratia*, and *Stenotrophomonas.* The exact species were presented in Table 1.

Siderophore production was noted for 21 out of 29 isolates (Table 1).

#### 2.2.5. Germination of Seeds 

**Figure 8:**
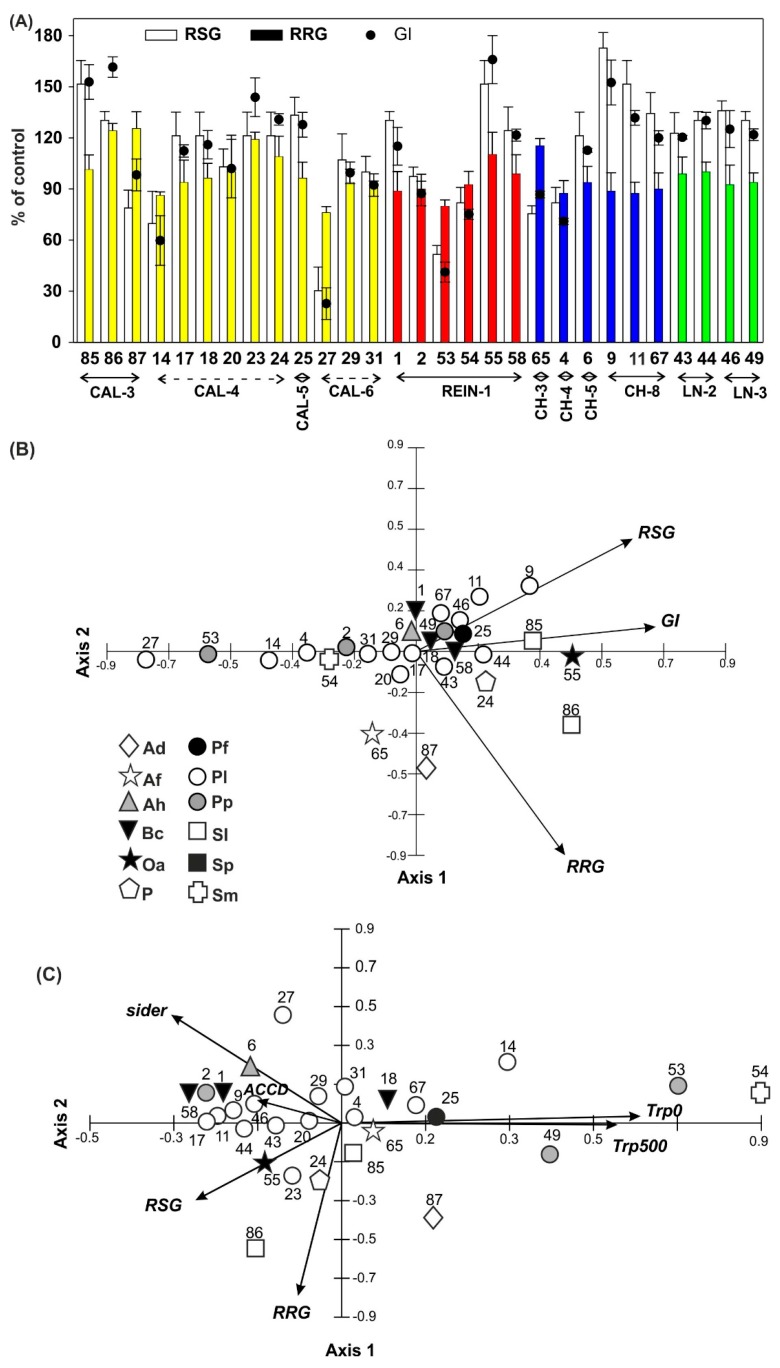
Seeds germination (RSG), root length (RRG), and germination index (GI) of *Phaseolus coccineus* (**A**). PCA for germination parameters: RSG, RRG, GI (**B**), and PCA for ACCD activity, IAA concentration with Trp0 and Trp500, RSG, RRG, and presence of siderophores (sider) (**C**). For other abbreviations, please see Table 1.

Germination results compared to control (Figure 8A) proved that relative seed germination (RSG) was higher in 20 isolates, relative root growth (RRG) rose in 7, and germination index (GI) was elevated in 19 isolates.

The PCA analysis focusing on the assessment of the impact of individual bacterial isolates on germination showed the statistical significance of all analyzed variables (Figure 8B). The first and second axes of the diagram explained 99.7% of the data variability, including axis 1 with 72.5%. The analyzed parameters were, in most cases, positively correlated with both axes of the diagram. GI showed the strongest relationship with the first axis and RSG with the second axis. Only RRG was negatively correlated with the second axis. The first axis of the diagram determined the gradient of influence of the bacterial isolates on seeds germination. On the left side of the diagram there were linearly arranged isolates 27, 53, and 14, with the lowest values of RSG and GI. Moving to the right of the diagram, the values of the above germination rates systematically increased and reached the maximum values for isolates 9, 55, 85, 11, and 46. RRG was partly inversely proportional to RSG. Then highest RRG values were assumed for isolates 87, 86, 23, and 65, with low or average RSG values.

#### 2.2.6. Relationship Among Bacterial Isolates 

Relationships among all tests conducted on bacterial isolates were analyzed in PCA (Figure 8C). PCA showed statistical significance of 5 out of 6 variables related to the first axis of the control diagram and 4 to the second axis. The first two axes explained 58.2% of the data variability, including the first one at 37.6%. With the first axis, Trp/500 and Trp/0 were positively correlated, whereas ACCD activity, RSG value, and the number of siderophores were negatively correlated. The negative relationship with the second axis was demonstrated by both germination rates, RSG and RRG, and the positive relationship by siderophore production and ACCD activity. ACCD activity and IAA concentration (Trp/0 and Trp/500) were correlated with each other. The highest IAA values for isolates 54, 49, 67, 53, and 14 were characterized by low ACCD values, as well as low germination rates. The occurrence of siderophores in isolates 58, 6, and 27 was associated with high ACCD activity, IAA production, and low and medium RRG values. In the case of isolates 9, 11, 55, and 46, the occurrence of siderophores was associated with high RSG values.

### 2.3. Relationship among Microbial Parameters Measured for Soil Samples and Bacterial Isolates

**Figure 9:**
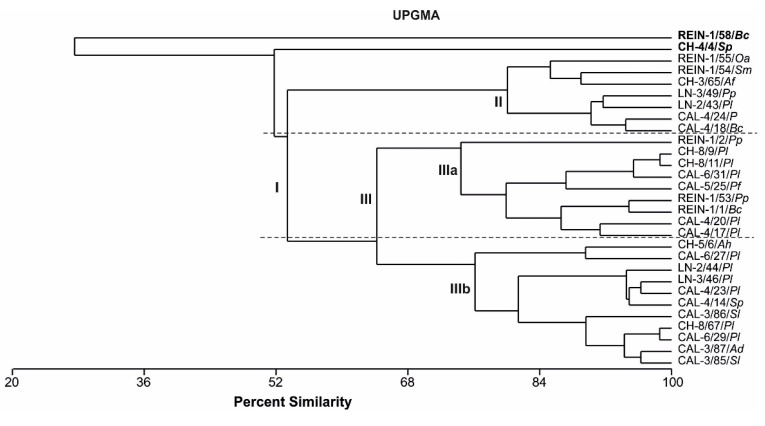
Dendrogram of the unweighted pair-group method with arithmetic mean (UPGMA) cluster analysis of 29 soil bacteria isolates, using a standardized percent similarity index. The analysis was performed on the basis of number of Copio and Oligo, SSB and PSB, AWCD index, DH activity, ACCD activity, IAA concentration with Trp0 and Trp500, and GI. For other abbreviations, please see Table 1.

The hierarchical cumulative classification was developed on the basis of quantitative characteristics of soil samples (number of copio- and oligotrophic bacteria, SSB, PSB, AWCD value, and DH activity) and bacterial isolates (ACCD activity, IAA production without and with Trp, GI, and siderophore production) (Figure 9). The dendrogram clearly showed the differences between the REIN-1/58/Bc and CH-4/4/Sp isolates and the remaining 27 samples (group I, 27.6% similarity). REIN-1/58/Bc isolate was distinguished by the highest level of ACCD, DH, and high values of Oligo_CFU and Copio_CFU in comparison with other samples. Conversely, CH-4/4/Sp isolate was characterized by the lowest values of the above mentioned parameters. The remaining 27 isolates formed group I with a similarity of 53.4%. They were divided into 2 groups, II and III, with similarity of 80.1% and 64.6%, respectively. Group II gathered isolates described by a very high and high number of bacteria and AWCD values, while showing low ACCD activity. As it was non-homogeneous, group III was divided into 2 groups, IIIa and IIIb, with similarity of 74.4% and 76.1%, respectively. Group IIIa accumulated samples characterized by average levels of ACCD and DH activity. Elevated CFU values for oligo- and copiotrophs were noted only for CAL-4/17/Pl and CAL-4/20/Pl isolates. In 8 out of 9 isolates grouped in IIIa, the production of siderophores was recorded. The values of other parameters were quite diverse. Group IIIb was characterized by high ACCD activity, but was definitely lower than for REIN-1/58/Bc, and showed low DH activity. Other indices were very diverse, e.g., raised CFU values with elevated AWCD level for both groups of bacteria were reported for CAL-4/14/Sp, CAL-4/23/Pl, CH-5/6/Ah, LN-2/44/Pl, and LN-3/46/Pl.

## 3. Discussion

Our experiments proved that permafrost microorganisms were not mainly quiescent, but led active metabolism, which is in accordance with other research [2]. We demonstrated that λ value for all tested soil bacterial populations was less than 0.5 day^−1^ (Figure 1B), therefore, soil populations were in the quiescent state, but not in the starvation state, which complies with Hattori [29,30]. Such λ values were sufficient for metabolic activity, which was confirmed by traits of PGPB isolates. Only 4 out of 17 oligotrophic bacteria with λ value between 0.02 and 0.05 could be classified as moving into a starved state, but the lowest value was 2.5 times higher than the exact starved state (λ~0.005). Generally, Tr parameter was higher for oligotrophs than copiotrophs. The increase of Tr (CAL-4, CAL-5, REIN-2, and LN-1 for oligotrophic bacteria, and CAL-7, REIN-2, CH-6, and CAL-5 for copiotrophic bacteria) (Figure 1C) may result from the quiescent state of cells. The lag time of their growth may be increased, and the decrease in the following period may reflect a partial refreshment of cells through coordinated expression of cannibalism and matrix production in the same subpopulation [30,31]. The outstanding small values of Tr in LN-2, CAL-7 for oligo-, and LN-3 for copiotrophic bacteria may imply that the population consists of subpopulations of different λ values. On the other hand, larger λ value may be explained, as some cells are nourished by the exudate from others, and the portion of nourished cells in the whole population may increase as the number of cells decreases [30], which could be seen in CAL-1, CAL-5, CAL-6, REIN-2, CH-2, and CH-4 (Figure 1A,C). Based on the PCA results (Figure 1D), despite correlation between number of copio- and oligotrophic bacteria, various correlations of the λ and Tr indices were observed for both groups of bacteria, which was the evidence for their physiological diversification.

Cellulase, amylase, and urease activities are quite commonly monitored among bacteria and have attracted much interest because of their diverse application for different purposes in industry [32], and depict additional benefits from plant growth stimulation. Our research showed that some soil samples represented a similar activity of all 3 enzymes, e.g., CAL-4, CAL-6, CH-4, and CH-8 (Figure 1C–E), whereas some seemed to be more specific for one prevalent activity. Among PGPB, not only isolates of multi-activities, but also of single activities could be important.

The EcoPlate analysis of soil microbial metabolic activity associated with the number of C substrates shows physiological potential of the dominant and culturable members of the bacterial community in comparison to soil real state [24,33]. In our research (Figure 4A,G), regardless of the indices used (especially similar results between pairs AWCD–NUSE, H–D, and opposite values for D–E, also depicting correlation), quite similar responses were achieved, exposing soils of weaker parameters (CAL-1, CAL-3, REIN-2) and stronger ones (LN-1, LN-2, CAL-4, REIN-1). Moreover, parameter D was inversely proportional to E. It seems that the observed responses of microorganisms could be both habitat and biochemically dependent. Among 9 substrates with high utilization rates (Figure S1), 7 were the same as presented by Kenarova et al. [26], and they could be assumed to be the most preferable nutrient sources for soil bacterial communities from cold regions. Moreover, according to Campbell et al. [34], among preferably utilized substrates were constituents of root exudates, such as L-asparagine and D-xylose, which could be useful in plant-microorganism interaction (Figure S1).

DH activity, sensitive to environmental changes, is often used as the indicator of soil fertility and health and it can denote the amount and activity of soil microbes [35]. DH activity is closely related to the carbon and nitrogen cycles and biological oxidation of soil organic matter, therefore its elevation was detected in good quality soils, and the lowest values were in poor quality acidic soils [36,37]. Our research did not prove the strong dependence between pH of soil [25] and DH activity (Figure 5). Increase in DH activity (LN-3, REIN-1, CAL-4) may represent and stimulate soil microbial activity and number (Figure 1A), and as a result increase the quantities and qualities of nutrients available to plants, demonstrated in the elevation of AWCD and catabolic diversity presented by NUSE, H, or R indicators (Figure 4, Figure 5B, Figure 8C), which was also observed by He et al. [38]. 

Plant rhizosphere is known to be a vast ecological niche for soil microorganisms representing various types of activity: induction of plant resistance in the presence of biotic and abiotic stress, the synthesis and release of siderophores [1], and phosphate solubilization [11] under Fe and P deficiency in the soil, respectively. Siderophores bind to the available form of iron Fe^3+^ in the rhizosphere, making it unavailable to the phytopathogens, protecting plant health and promoting plant growth [1]. In the present investigation, SSB were detected in all soil samples and 72% of bacterial isolates synthesized siderophores (Table 1). Almost all bacteria classified as *Pseudomonas* possessed the ability to produce SSB [39,40]. The PCA diagram (Figure 5B) clearly depicted reverse dependence between the number of SSB and the concentration of available Fe in the soil. 

PSB may transform various P insoluble compounds into soluble ones available for plants [33] and their number detected in all examined soil samples was quite high. Nevertheless, as seen in Figure 5B, fluctuations between the number of PSB and P content in soil were varied, without showing one clear tendency. Inorganic P solubilization and organic P mineralization can coexist in the same bacterial strain [41]. Solubilization of inorganic phosphates is caused by low molecular weight acids (e.g., gluconic and citric acids) synthetized by soil bacteria, but organic phosphate mineralization occurs through synthesis of phosphatases by bacteria, which catalyze the hydrolysis of phosphoric esters, releasing the phosphate group [9]. Moreover, from our research, it seemed obvious that siderophore production and phosphate solubilization co-occur, but the strength of action was mainly oppositely pronounced (e.g., in CAL-3 (Figure 2A,B)), which means that soil samples were dominated by bacteria with specified activity, thus a higher number of SSB was connected with a lower PSB number.

Promotion of plant growth by PGPB is often examined by their ability to produce ACCD and auxins [5]. ACCD in microorganisms is expressed on the availability of a signaling molecule, ACC, and is regulated differentially by various physiological factors [15,42]. ACCD-producing bacteria were more abundant in the rhizosphere of plants growing under stressful conditions (e.g., heavy metal contamination, cold stress) than in non-stressful conditions [43], indicating a selection of bacteria under averse environments that is consistent with the increased ET and ACC levels induced by heavy-metal or cold stresses [17,44]. Moreover, ET (with JA and salicylic acid), as a signaling molecule, is a component of systemic resistance [14]. On the basis of the above mentioned statement, the level of ACCD activity in our experimental samples predominantly showed that microorganisms were growing under stressful environmental conditions (e.g., isolates numbers 58, 6, 27, 29, 67, 87, 85, 23, and 46) and were similar to levels demonstrated by Acuña et al. [45]. In contrast with Zhao et al. [46], we did not detect correlation between pH of the soil and the highest ACCD activity, but in our samples the pH was lower, being in the range from 7.8 to 5.1, and did not exceed 8.0 [25]. Therefore, we have not detected strong soil alkalization, which occurs as a consequence of changes of ion fluxes across the plasma membrane caused by rapid production of ACC and ET [47]. 

Root growth inhibition by ET is a common adaptation in order to avoid adverse agents [48]. Therefore, it can also be assumed that bacterial isolates with low ACCD activity (numbers 55, 49, 43, 54, 65, and 24) could be potentially useful in plant growth stimulation. Co-evolution of plants with ethylene-reducing microorganisms, such as ACCD producing bacteria, may generate elevated ACC production to supplement microbial degradation [16,48]. Bacterial isolates examined in our experiment showed ACCD levels generally smaller than those stated by Kumar et al. [49]. 

IAA stimulates cell elongation, division, and differentiation in plants [6], and is responsible for the process of plant-microorganism interaction [18]. About 80% of rhizospheric bacteria possess ability to produce IAA, which is in accordance with the results presented by Souza et al. [10]. Only 5 (including 1 very weakly producing IAA) out of 29 isolates were unable to intrinsically synthesize IAA without Trp supplementation (Figure 6). Based on the PCA results (Figure 7B), addition of Trp caused elevation of IAA production, but mostly this was proportional to the data achieved without Trp addition. The difference in bacterial efficiency to IAA synthesis resulted mainly from the number of bacteria. 

For stimulation of IAA biosynthesis, we have supplemented the microbial cultures with L-Trp, which was shown to be the most effective IAA precursor from any other isomeric forms of Trp, e.g., DL or D [50]. Depending on the pH of the Spitsbergen soil, different forms of IAA can be expected. In weakly acidic rhizosphere (REIN-1, CH-3, 4, 5, 8, and LN-2 and 3) [25], a substantial part of IAA might be protonated to be able to enter inside the bacteria by membrane diffusion (based on the membrane permeability and pH) [51]. However, in neutral and basic pH (CAL-3, 4, 5 and 6) [25], a prominent amount of IAA may be in anionic form to be able to get into bacteria by action of a proton-driven influx carrier [52].

Moreover, based on the fact that the IAA biosynthesis in bacteria is dependent on the concentration of Trp taking the optimal level at 500 µg mL^−1^ [53], we have chosen exactly this concentration in our research. We have demonstrated that the synthesis of IAA was also isolate-type-dependent, which was in accordance with other studies [53]. The concentration of IAA produced by microorganisms seems to be related with plant-type rhizosphere, reaching values from 35.31 to 43.08 µg mL^−1^ IAA for corn and rice rizospheric phosphobacteria, respectively [54], and with microorganism types, not exceeding 21 µg mL^−1^ IAA, even after 500 µg mL^−1^ Trp supplementation (Figure 6). Our data were in accordance with other studies on *Pseudomonas* strains or isolates, which after 500 µg Trp mL^−1^ application synthetized from 18.07 to 22.02 µg IAA mL^−1^ [53,55].

The optimal IAA concentration for plants may be narrow, thus up to 10^−8^ M can stimulate plant growth [56], 1 × 10^5^ CFU mL^−1^ has no effect on plant, but 1 × 10^6^ CFU mL^−1^—inhibits plant growth [57]. There is evidence that production of IAA alone does not guarantee root elongation or growth promotion [58]. Our germination test showed the above mentioned correlation because the highest concentration of IAA without Trp supplementation in isolates numbers 54 and 53 (Figure 6) caused reduction in RSG, RRG, and GI parameters (Figure 8), compared with most of the isolates with lower levels of IAA. We agree that such findings may have direct practical application, although ability of bacteria to produce IAA depends on the availability of precursors and uptake of microbial IAA by plant [53]. Strains overproducing IAA can also be used as Biological Control Agents, e.g., strains might serve as weed pathogens, especially those attacking agriculturally important crops [6]. Approximately 69% of isolates increase RSG, which corresponds with GI (66%), number of isolates able to produce siderophores (72%) and IAA (83%), and with weaker correlation with ACCD activity.

In agreement with the statement that auxin and its signaling mechanism are necessary for the ACC and ET-induced root elongation inhibition [59], we confirmed that generation of IAA (Figure 6) lowered ACCD activity (Figure 7), elevated IAA concentration (Figure 6), and yielded reduction of root growth (Figure 8), as seen in isolates numbers 14, 25, 53, 54, 67, and 49, among others. However, isolates with corresponding high IAA and ACCD values were also observed. Acuña et al. [45] demonstrated similar variations between IAA and ACCD levels. 

Results of phenotypic identification by API tests used in screening of PGPB seem to be reliable when compared with other methods of identification, such as the automated instrument for microbial identification and antibiotic susceptibility testing system, VITEK 2 fluorescent system and 16S rRNA gene sequencing [60].

The source of PGPB might also be soils of lower biological activity. Some of the above-tested isolates could exhibit multiple traits, which may promote plant growth directly, indirectly, or synergistically, which is in accordance with other findings [5]. Not all of the mentioned mechanisms need to appear in one bacteria species to be able to have measurable effects on plant physiological status because also other factors (e.g., metabolites) could contribute to the final effect on plant physiology [13]. In order to apply new PGPB to plant culture, the appropriate inoculum density should be considered to avoid negative dosage-dependent influence on nutrient supplies [5].

Findings achieved from soil and isolate analyses presented in the dendrogram (Figure 9) depict great diversity of soil samples collected from Spitsbergen in terms of biological activity and traits of isolates crucial for features promoting growth. In our research, GI was a more accountable factor in comparing the results of germination instead of RSG or RRG alone, similar to the results of Pampuro et al. [61]. In our research *Pseudomonas* genus also seems to be promising for future experiments because of its plant growth promoting potential, as was demonstrated by others [3,55].

## 4. Materials and Methods 

### 4.1. Description of the Study Area and the Soil Samples 

The study area was situated on the largest island of the Svalbard Archipelago in the High Arctic, i.e., in Spitsbergen. Precisely, it was located in the SW area of Spitsbergen, the NW part of Wedel Jarlsberg Land, in Recherchefjorden.

The soil samples were collected in 32 distinct areas (Table 2) from close vicinity of the plant root system. Between July and August 2014, in each area, five soil samples were collected from a depth of 15 cm, mixed together, sieved under sterile conditions, and used in the experiments. Further description of the study area and soil samples can be found in Hanaka et al. [25]. 

### 4.2. Soil Analysis

#### 4.2.1. Copio- and Oligotrophic Bacteria Enumeration

The heterotrophic bacterial communities (cophiotrophs and oligotrophs) inhabiting the soils were analyzed using the agar plate method on the media described in Hanaka et al. [25]. The colony formation by soil bacteria was determined by the FOR probabilistic model proposed by Hattori [29], which permitted assessment of the physiological state of the cells that had inhabited the initial soils of Spitsbergen. Colonies were counted on the agar plates each day for 21 days. Three parameters characterizing the kinetics of colony formation were determined using the following equation [31,62]: N_(t)_=N_inf_{l − exp[−λ(t − Tr)]}, where N_(t)_ and N_inf_ are the numbers of colonies observed at time t and the infinite time (first parameter), Tr, retardation time, shows the interval between plating and the initiation of colony appearance, thus reflecting the growth rate (second parameter), and λ indicates probability of visible colony formation per unit time (third parameter). This model assumes that bacterial cells form visible colonies at a probability of cell proliferation λ (day^−1^) after a Tr period (days), and also, that bacterial cells existing in soil microbial community manifest three physiological states: when λ < 0.5 bacteria are in quiescent phase (going into starvation phase at λ = 0.2 day^−1^ or lower value) [30]; 0.5 < λ > 1 – in transient phase; λ > 1 – in vegetative phase.

#### 4.2.2. Determination of Number of Specific Soil Bacteria 

Siderophore production by SSB was tested on the Chrome azurol S agar medium [63] and a yellow-orange halo around the growth area was observed. This procedure was conducted first for soil dilutions, then for isolated bacterial strains. The number of PSB showing a halo effect around the colony was counted on Pikovskaya [64] modified medium with the following components: glucose 10 g; Ca_3_(PO_4_)_2_ 5 g; (NH_4_)_2_SO_4_ 0.5 g; NaCl 0.2 g; KCl 0.2 g; MgSO_4_ × 7 H_2_O 0.1 g, MnSO_4_ × H_2_O 0.002 g; FeSO_4_ × 7 H_2_O 0.002 g; agar 15 g; 1000 mL deionized water.

On medium containing a 1% (*w*/*v*) solution of carboxymethylcellulose (CMC) [65], cellulolise areas were elicited by 0.1% Congo Red dye followed by 1 M NaCl, both of which were applied for 20 min [66], which visualizes activity of CB. To identify bacteria with amylase activity, known as AB, the isolates were cultivated on the agar medium with the following components: starch 10.0 g; KH_2_PO_4_ 0.5 g; MgSO_4_ × 7 H_2_O 0.5 g; (NH_4_)_2_SO_4_ 0.2 g; agar 15 g; 1000 mL deionized water. Plates with bacterial colonies were flooded with 0.01 M J_2_-KJ solution. AB showed a clearing halo around the growth area. Media containing urea [67] were applied for detection of UB, which gave a dark blue color. King’s B medium [68] specific for *Pseudomonas* detection was used for fluorescence observations under UV exposure and the strains grown on this medium were further examined in API tests. 

All media with soil dilutions or bacterial isolates on the plates were incubated at 20 °C and the number of colonies was counted every day for 14 days, then depending on the procedure, halo areas or dyed colonies were counted.

#### 4.2.3. Analysis of the Community-Level Physiological Profiling

The community-level physiological profiling (CLPP) or metabolic fingerprint was determined by Biolog EcoPlate assay (Biolog Inc., Hayward, CA, USA) to characterize and classify heteropteric microbial communities based on carbon source utilization patterns [33]. The plates contained 31 sources of carbon [2,24], including components of exudates of plant roots [34]. Briefly, soil was shaken in distilled sterile water and 10^−3^ soil dilution was inoculated and incubated at 20 °C. Optical density was taken every 24 h for 240 h at 590 nm. The microbial community response was detected as the production of NADH caused by cell respiration [33]. CLPP was evaluated on the basis of utilization of carbon substrate and presented with appropriate indexes. 

#### 4.2.4. Determination of Soil Microbial Activity and Catabolic Diversity

To assess the general activity and physiological function of microbial communities by Biolog EcoPlate, AWCD was calculated using the following equation: AWCD = Σ(n_i_ − c)/31, where n_i_ and c were the average absorption at 590 nm of each well of the substrate and the control well (without a C source), respectively, and 31 is the number of carbon sources in the EcoPlate [26]. AWCD was calculated daily during 10-day incubation and the kinetic curves were presented (Figure 2), as well as score at endpoint (Figure 3).

To compare the catabolic diversity of the microbial community among different soil samples, H, R, E [69], D [70], and NUSE [71] were calculated and shown in Figure 3. H index was calculated as H = −Σ *p*_i_(ln*p*_i_), where *p_i_* is the ratio between the relative value of absorption (n_i_ − c) of the substrate and the sum of the entire plate [72]. R refers to the total number of different carbon sources metabolized by the soil microbial community. It is expressed as the number of wells whose OD_590_ value (n_i_ − c) was > 0.06 in each plate, as Wang et al. [23] recommended. E was calculated as E = H/lnR [35]. D was expressed as follows: D = Σ (N_1_/N)^2^, where N_1_ is the OD_590_ value of each substrate individually and N is the sum of OD_590_ values of all utilized substrates [70]. NUSE index was expressed as % of the summed OD_590_ of substrates containing nitrogen in the total OD_590_ of all utilized substrate [71].

#### 4.2.5. Analysis of Dehydrogenase Activity

The DH activity was determined by the reduction of 2,3,5-triphenylotetrazolium chloride to triphenyl formazan according to Casida et al. [73] and Alef and Nannipieri [74]. This analysis shows the positive answer at neutral range of pH and in presence of CaCO_3_ for buffering soil system, so the soil samples were prepared as described by Januszek et al. [75].

### 4.3. Bacterial Activity

#### 4.3.1. ACC Deaminase Activity

The ACCD activity was assayed according to a modified method of Belimov et al. [76] and Shaharoona et al. [16] by measuring the amount of α-ketobutyrate, a product released after hydrolysis of ACC. All incubations were processed at modified temperature of 25 °C. For further calculations, the protein concentration was quantified by the Bradford method [77].

#### 4.3.2. Analysis of IAA Synthesis

The ability of the bacterial isolates to produce IAA was tested on Czapek-Dox modified medium (1% glucose), pH 7.0, with an addition of 500 µg Trp, IAA precursor. Bacterial cultures were incubated at 20 °C for 24 h. The concentration of IAA in liquid culture was measured using the Salkowski reagent [78] and estimated against the standard curve of IAA (Sigma-Aldrich, St. Louis, MI, USA) [19].

#### 4.3.3. MIC for Cu

A full loop of bacterial suspensions (about 1 × 10^5^ CFU mL^−1^) was placed on the nutrient agar, pH 7.0, with an addition of sterilized CuSO_4_ × 5 H_2_O (1; 5; 10 and 15 mM) and observed for 14 days. MIC was determined as the lowest concentration of a chemical, which prevents visible growth of bacterium.

#### 4.3.4. Biochemical Identification of Bacteria 

For biochemical identification of bacteria, two types of API tests were used. API 20 NE V8.0, as a mixture of classical and assimilative tests, is dedicated for identification of non-fastidious isolates of aerobic, non-fermenting, Gram-negative, non-Enterobacteriaceae rods. API 50 CHE V3.2, as a performance of carbohydrate metabolism tests, was committed for identification of Gram-positive bacteria, as well as Gram-negative bacteria belonging to the *Enterobacteriaceae*. Testing was conducted according to the instructions of the manufacturer (bioMérieux Inc., Marcy-l’Etoile, France). Automated interpretation of API strip results was done after 24 and 48 hours using the identification software, with the database at https://apiweb.biomerieux.com. 

#### 4.3.5. Seed Germination Test

Culture of runner bean (*Phaseolus coccineus*) seeds in the presence of isolates was conducted in vertical plastic containers according to the instructions of the Phytotoxkit microbiotest manufacturer (MicroBioTests Inc., Mariakerke, Belgium). Briefly, after seed sterilization was conducted in 5% H_2_O_2_, they were inserted into the containers filled with sterylized soil. Next, on the seed surface, the bacterial isolates (1 × 10^7^ CFU g^−1^ DW of soil) were applied. The number of germinated seeds and length of roots were measured after 120 hours of exposure to isolates and compared to control (non-isolate treated). On this basis, three parameters were calculated: (1) RSG (%) = S_N_/S_C_ × 100, where S_N_ is the mean number of seeds germinated in isolate environment and S_C_ is the mean number of seeds germinated in control; (2) RRG (%) = R_N_/R_C_ × 100, where R_N_ is the mean root length grown in isolate environment and R_C_ is the mean root length in control; and (3) GI (%) = (RSG × RRG)/100 [61].

### 4.4. Statistical Analysis

The statistical analyses were conducted using Statistica 12.5 (Stat Soft. Inc.) and were presented as the mean value $\bar{X}$ with standard deviation (SD). The statistical multivariate methods were evaluated using the MultiVariate Statistical Package (MVSP) 3.21 package [79]. The data were assayed after standardization. Based on the length of the data gradient (0.8–1.59 SD), PCA analysis was carried out. All analyses were based on the average values of variables with statistical significance of *p* < 0.05 for a given sample. The similarity of the soil bacteria isolates, in particular study areas, was estimated using percent similarity index and cluster analysis performed by MVSP 3.21. The analysis of similarities was performed using the unweighted pair-group method with arithmetic mean (UPGMA).

## 5. Conclusions

Bacterial isolates from Spitsbergen soils possess diverse characteristics, which could be useful for various goals. The differences between λ value for copio- and oligotrophic bacteria were proved. High DH activity was accompanied by elevated number of copio- and oligotrophic bacteria, as well as raised AWCD value and N and C contents in the soil. In most cases, a high number of SSB accompanied an elevated number of PSB. A raised number of SSB was strongly connected with low values of Fe in the soil. Bacterial isolates were efficient in IAA synthesis, with the level of efficiency increased after Trp supplementation. GI was more similar to RSG than RRG. Parameters such as GI, number of isolates able to produce siderophores and IAA, and to a lesser extent ACCD activity corresponded with the number of isolates provoking increase in RSG. ACCD activity lowered with elevation of number of copio- and oligotrophic bacteria and with IAA concentration. Synthesis of siderophores was matched with ACCD activity and its high level was combined with elevated GI. In spite of the different localization of soil samples, some isolates proved similar traits of activity. Distinct affiliation of isolates and their various localizations were displayed. PGPB have a significant contribution in pioneering conditions, which prevail in Spitsbergen.

PGPB are a part of rhizosphere microbiome, which is crucial for plant resistance maintenance in stress and pioneering conditions. Deep exploration of efficient PGPB, which can colonize plants effectively and increase plant productivity under different conditions, will uncover their mechanism of action and complete potential. The potential of these isolates in a pot experiment should be undertaken, especially on *Pseudomonas* genus isolates, which have a broad desirable profile of activities.

## Figures and Tables

**Table 1:** Identification of strains according to the analytical profile index (API) tests and their ability in siderophore synthesis. All strains are Gram-negative, otherwise * shows Gram-positive isolate.

Soil Sample	Isolate No	Bacteria Genus/Species	Bacteria Abbreviation	ID [%]	Siderophore Synthesis
CAL-3	85	*Serratia liquefaciens*	*Sl*	96.3	No
CAL-3	86	*Serratia liquefaciens*	*Sl*	95.9	No
CAL-3	87	*Achromobacter denitrificans*	*Ad*	82.2	No
CAL-4	14	*Serratia plymuthica*	*Sp*	99.9	Yes
CAL-4	17	*Pseudomonas luteola*	*Pl*	92.5	Yes
CAL-4	18	*Burkholderia cepacia*	*Bc*	67.8	Yes
CAL-4	20	*Pseudomonas luteola*	*Pl*	92.5	Yes
CAL-4	23	*Pseudomonas luteola*	*Pl*	92.5	Yes
CAL-4	24	Pantoea spp 1*	*P*	82.3	No
CAL-5	25	*Pseudomonas fluorescens*	*Pf*	99.9	Yes
CAL-6	27	*Pseudomonas luteola*	*Pl*	92.5	Yes
CAL-6	29	*Pseudomonas luteola*	*Pl*	99.8	Yes
CAL-6	31	*Pseudomonas luteola*	*Pl*	99.8	Yes
REIN-1	1	*Burkholderia cepacia*	*Bc*	67.8	Yes
REIN-1	2	*Pseudomonas putida*	*Pp*	87.9	Yes
REIN-1	53	*Pseudomonas putida*	*Pp*	89.1	No
REIN-1	54	*Stenotrophomonas maltophilia*	*Sm*	99.9	No
REIN-1	55	*Ochrobactrum anthropi*	*Oa*	95.9	Yes
REIN-1	58	*Burkholderia cepacia*	*Bc*	67.8	Yes
CH-3	65	*Alcaligenes faecalis 1*	*Af*	90.1	Yes
CH-4	4	*Serratia plymuthica*	*Sp*	99.9	No
CH-5	6	*Aeromonas hydrophila*	*Ah*	99.7	Yes
CH-8	9	*Pseudomonas luteola*	*Pl*	99.8	Yes
CH-8	11	*Pseudomonas luteola*	*Pl*	99.5	Yes
CH-8	67	*Pseudomonas luteola*	*Pl*	99.8	Yes
LN-2	43	*Pseudomonas luteola*	*Pl*	99.8	Yes
LN-2	44	*Pseudomonas luteola*	*Pl*	99.5	Yes
LN-3	46	*Pseudomonas luteola*	*Pl*	99.8	Yes
LN-3	49	*Pseudomonas putida*	*Pp*	99.1	No

**Table 2:** The sampling sites of NW part of Wedel Jarlsberg Land (Spitsbergen, Svalbard) with plant species occurring in particular areas. Abbreviations: CAL = Calypsostranda; REIN = Reinholmen; CH = Chamberlindalen; LN = Lӕgerneset; Dra = *Draba corymbosa*; Dry = *Dryas octopetala*; Sal = *Salix polaris*; Sax = *Saxifraga oppositifolia*; ns = plant communities not determined due to the absence of dominant species.

Soil Sample	Area	Coordinates		Dominant Plant Species	C ^1^	N ^1^	C/N ^1^	P ^1^	Fe ^1^
		N	E		%			mg kg^−1^	g kg^−1^
CAL-1	CAL	77°34′10″	14°25′26″	Sax	4.37 ± 0.22	0.052 ± 0.003	84.04	3.44 ± 0.49	0.61 ± 0.08
CAL-3		77°33′57″	14°28′32″	Dra	3.50 ± 0.17	0.105 ± 0.013	33.33	1.09 ± 0.11	0.52 ± 0.04
CAL-4		77°33′55″	14°28′57″	Dry	5.19 ± 0.12	0.394 ± 0.036	13.17	1.99 ± 0.16	0.40 ± 0.02
CAL-5		77°33′55″	14°28′57″	Dry	5.55 ± 0.04	0.381 ± 0.013	14.19	3.08 ± 0.15	0.65 ± 0.07
CAL-6		77°33′55″	14°28′57″	Sax	4.31 ± 0.01	0.310 ± 0.020	13.90	1.81 ± 0.14	0.74 ± 0.15
CAL-7		77°33′59″	14°29′41″	Dra	2.40 ± 0.19	0.075 ± 0.009	32.00	10.70 ± 1.05	0.34 ± 0.01
REIN-1	REIN	77°29′45″	14°33′17″	Dry	3.15 ± 0.20	0.202 ± 0.036	15.59	19.04 ± 1.95	0.29 ± 0.04
REIN-2		77°29′45″	14°33′20″	ns	25.42 ± 1.49	1.253 ± 0.103	20.29	40.43 ± 2.11	0.70 ± 0.01
CH-2	CH	77°29′00″	14°31′48″	Dry	3.01 ± 0.13	0.218 ± 0.057	13.81	21.94 ± 1.40	0.42 ± 0.03
CH-3		77°29′00″	14°31′51″	ns	0.88 ± 0.06	0.079 ± 0.005	11.14	4.89 ± 0.31	0.36 ± 0.02
CH-4		77°28′59″	14°31′53″	Sal	1.15 ± 0.12	0.116 ± 0.023	9.91	95.36 ± 4.23	0.26 ± 0.01
CH-5		77°28′59″	14°31′52″	ns	1.81 ± 0.14	0.148 ± 0.012	12.23	6.35 ± 0.51	0.31 ± 0.02
CH-6		77°28′52″	14°32′01″	ns	2.85 ± 0.29	0.235 ± 0.035	12.09	13.05 ± 1.27	0.27 ± 0.07
CH-7		77°34′10″	14°25′26″	ns	1.25 ± 0.41	0.145 ± 0.006	8.62	3.99 ± 0.40	0.39 ± 0.08
CH-8		77°34′04″	14°28′05″	ns	4.13 ± 0.26	0.399 ± 0.025	10.33	11.24 ± 0.72	0.31 ± 0.02
LN-1	LN	77°33′55″	14°28′57″	Dry	19.42 ± 0.24	0.883 ± 0.077	21.99	7.61 ± 0.69	0.37 ± 0.08
LN-2		77°33′55″	14°28′57″	ns	12.62 ± 0.50	0.356 ± 0.032	35.45	4.71 ± 0.11	0.60 ± 0.01
LN-3		77°33′59″	14°29′41″	ns	15.23 ± 0.59	0.628 ± 0.055	24.25	13.96 ± 0.49	0.58 ± 0.08

^1^ Hanaka et al. [25].

## Supplementary Materials

Supplementary materials can be found at https://www.mdpi.com/1422-0067/20/5/1207/s1.

## Abbreviations

PGPB	Plant growth promoting bacteria
CB	Cellulolytic bacteria
AB	Amylolytic bacteria
UB	Ureolytic bacteria
SSB	Siderophore synthesizing bacteria
PSB	Phosphate solubilizing bacteria
ACCD	ACC (1-aminocyclopropane-1-carboxylate) deaminase
IAA	Indole-3-acetic acid
AWCD	Average well color development
Trp	Tryptophan
CLPP	Community-level physiological profiling
H	Shannon’s diversity index
R	Substrate richness
E	Substrate evenness
D	Domination index
NUSE	Nitrogen use index
MIC	Minimal Inhibitory Concentration
CAL	Calypsostranda
REIN	Reinholmen
CH	Chamberlindalen
LN	Lӕgerneset
