# Supplementary material for: Physiological Diversity of Spitsbergen Soil Microbial Communities Suggests Their Potential as Plant Growth-Promoting Bacteria
Source: Int J Mol Sci. 2019 Mar 9;20(5):1207. doi: 10.3390/ijms20051207 (PMC6429280; doi:10.3390/ijms20051207)
Supplement: Supplementary file 1 [file ijms-20-01207-s001.pdf]

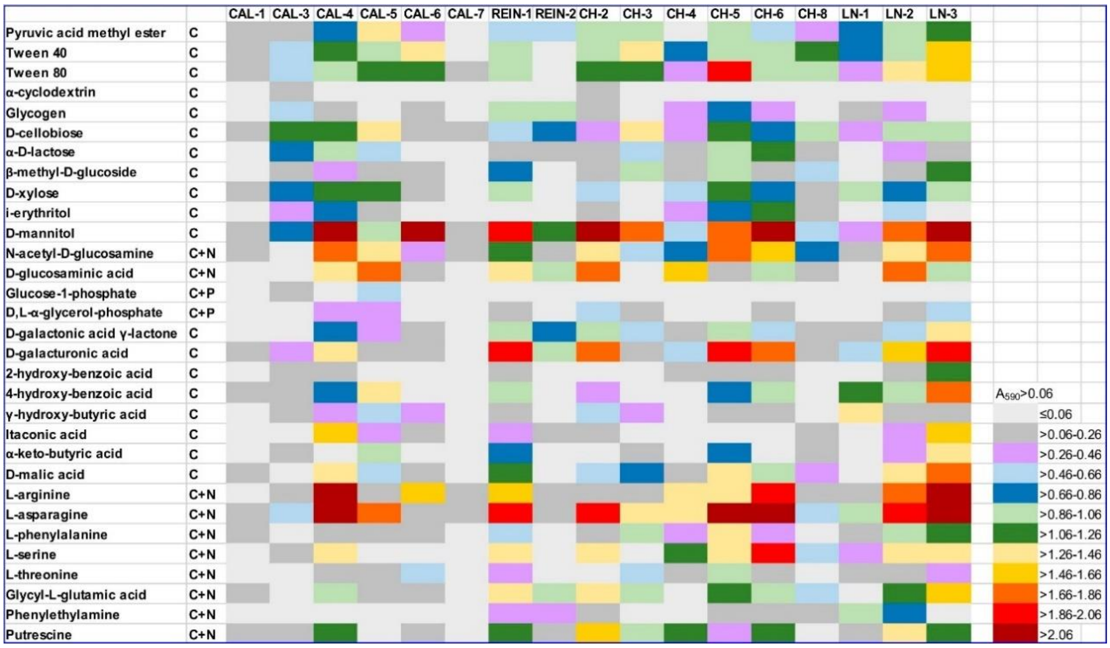

Figure S1. Heat map for the carbon utilization patterns of the substrates applied in the Biolog EcoPlates after 10 days of soil incubation.
